# Frequency of hematologic and solid malignancies in the family history of 50 patients with acute myeloid leukemia – a single center analysis

**DOI:** 10.1371/journal.pone.0215453

**Published:** 2019-04-18

**Authors:** Anne-Sophie Sandner, Ramona Weggel, Yasmin Mehraein, Stephanie Schneider, Wolfgang Hiddemann, Karsten Spiekermann

**Affiliations:** 1 Department of Medicine III, University Hospital Munich, Ludwig-Maximilians-University Munich—Campus Großhadern, Munich, Germany; 2 Institute of Human Genetics, University Hospital Munich, Ludwig-Maximilians-University Munich, Munich, Germany; 3 German Cancer Consortium (DKTK), Heidelberg, Germany; 4 German Cancer Research Center (DKFZ), Heidelberg, Germany; 5 Clinical Cooperative Group Leukemia, Helmholtz Center Munich, Germany; Ohio State University Wexner Medical Center, UNITED STATES

## Abstract

**Background and objective:**

The revised World Health Organization classification of 2016 for myeloid neoplasms and acute leukemia added a section of myeloid neoplasms with germline predisposition. The main objective of our study was to evaluate the frequency of hematologic and solid malignancies in the family history of patients with acute myeloid leukemia (AML) by using a systemic pedigree interview. The family history was taken of 50 patients between 24 and 80 years.

**Findings:**

8/50 (16%) patients with AML had family members with hematologic malignancies. 2/50 (4%) patients had family members of first degree with hematologic malignancies. Furthermore in 42/50 (84%) of AML patients solid malignancies were documented in family members of any degree and in 31/50 (62%) in family members of first degree. The most commonly occurring malignancies in our cohort were breast and colorectal cancer. We analyzed the pedigrees for cancer syndromes that can be associated with acute leukemia like Li-Fraumeni syndrome, Lynch syndrome and hereditary breast cancer. 2/50 (4%) patients fulfilled the criteria for familial breast and ovarian cancer from the German consortium and 1/50 (2%) patients fulfilled the Bethesda Guidelines criteria for hereditary nonpolyposis colorectal cancer. No pedigree met the criteria for Li-Fraumeni syndrome. In 29 cases we compared the patient history obtained in the routine work-up with our data. The accuracy of the obtained family history was 23%, outlining that in the clinical routine information about family histories often escapes notice.

**Conclusion:**

Our study shows that though generally considered a sporadic disease, the presence of hematologic and solid malignancies in the family history of AML patients is relatively high. One should keep in mind that cancer syndromes like hereditary breast cancer are associated with a higher incidence of leukemia. These data are relevant in the context of family donor search for allogeneic stem cell transplantation, genetic counseling and testing as well as cancer prevention.

## Introduction

Myeloid neoplasms have long been considered to be sporadic diseases. In recent studies it could be demonstrated that there are several germline mutations that lead to a familial predisposition for acute myeloid leukemia (AML) and myelodysplastic syndrome (MDS) [1–3]. Many germline mutations are already defined, for example germline mutations in the *RUNX1*, *CEBPA* and *GATA2* gene. These findings have led to the implementation of a new category in the revised World Health Organization (WHO) classification of 2016 for myeloid neoplasms and acute leukemia namely myeloid neoplasms with germline predisposition as well as in the recent European Leukemia Network (ELN) classification named familial myeloid neoplasms. [4, 5]

Nevertheless, familial cases of AML and MDS are only seldom encountered in the clinical routine and the question remains if there are a relevant number of unidentified cases as the family histories were not taken elaborately enough.

The family history is an integral part of the patient history of cancer patients. The American Society of Clinical Oncology recommends as minimum family history for individuals with cancer to obtain information about first and second-degree relatives both on maternal and paternal sides, ethnicity, age at cancer diagnosis and type of primary cancer for each cancer case in the family. Moreover the minimum adequate cancer family history should encompass results of any cancer predisposition testing in any relative. This information should be taken at diagnosis and updated in the follow-up. [6]

There are several cancer syndromes that can be associated with the occurrence of leukemia, like Fanconi anemia [7]. For our cohort we focused on cancer syndromes that predispose for breast cancer and colon cancer, as they are two of the most frequent cancer entities and also most common in the pedigrees of our patients. Therefore we evaluated our data for the criteria for hereditary breast cancer with *BRCA1* or *BRCA2* mutations [8–10], Lynch syndrome/Lynch-mimic-syndrome [11–13] and Li-Fraumeni syndrome [14].

The aim of our study was to investigate the frequency of AML patients with a positive family history for hematologic and solid malignancies treated in our clinical center. Moreover we wanted to compare the accuracy of the family history obtained at initial admission in the clinical routine with our results.

## Materials and methods

From January 2016 until September 2017 we asked 50 unselected AML patients (de-novo-AML and secondary AML) older than 18 years to take approximately 30 minutes to obtain a detailed family cancer history. All patients were treated in our clinical center and signed informed consent. A comprehensive at least three-generation pedigree was obtained as broad as it was remembered by the patient. First-degree family relationships were parents, siblings and children. Second-degree relationships were uncles/aunts, nephews/nieces, grandparents/grandchildren and half-siblings and third-degree relationships were cousins. The definition of family relationship grades was applied according to the American Society of Oncology. [6] The pedigrees encompassed 3 to 5 generations and 6 to 47 family members per pedigree.

Additionally a standardized questionnaire [S1 Questionnaire] was handed out to the patient before obtaining the pedigree. In this questionnaire, specific questions concerning blood count changes, bleeding disorders and skin disorders in family members are listed, as these symptoms can hint to a familial predisposition for myeloid malignancies. [15]

All patients with an indication for a familial cancer predisposition were recommended to undergo genetic counseling and to suggest genetic counseling to their next of kin.

To evaluate how many families met the criteria for hereditary breast cancer and lynch syndromes, we applied the Bethesda or Amsterdam criteria for HNPCC and criteria for genetic testing of the German Consortium for familial breast cancer. [16–18]

Of the 50 patients in our study, 44 patients were initially diagnosed in our center and we were able to review the patients’ medical chart at initial admission. From 7 patients no patient history was taken at first admission due to intensive care admission or instable conditions and in 8 cases no family history was documented in the initial workup. Subsequently we compared the family history documented at initial admission with the obtained three-generation pedigrees of this study in 29 cases. Only 28% (8/29) of the family histories documented using the standard patient history form were completely accurate compared with the pedigree we obtained for this study.

The data were analyzed in a double pseudo-anonymized manner and statistical calculations were acquired with SPSS. Inclusion criteria were patients with diagnosis of AML, informed patient consent, sufficient German language skills, no cognitive deficits and an inpatient stay in the normal ward of our department for hematology and oncology. Changes in blood count were not defined as hematologic malignancies but classified as an own category.

The Ethics Committee of the University of Munich/LMU approved this study, which was performed according to the Declaration of Helsinki.

## Results

We analyzed the pedigrees of 50 AML patients from 24 to 80 years. Median age was 59.5 years, 32% of the patients were female. 72% of the patients were diagnosed with de-novo AML. Secondary AML arising from a myelodysplastic or myeloproliferative syndrome was diagnosed in 28% of the patients. [Table 1]

**Table 1:** Clinical and genetic characteristics of 50 AML patients.

Characteristic	Value
Total	50 (100%)
Sex	
male	34 (68%)
female	16 (32%)
Median Age	59.5 years
Age Groups	
20–30	4 (8%)
30–40	6 (12%)
40–50	6 (12%)
50–60	9 (18%)
60–70	16 (32%)
70–80	8 (16%)
80–90	1 (2%)
Diagnosis	
De novo AML	36 (72%)
Secondary AML	14 (28%)
Normal karyotype AML	20 (40%)
AML with biallelic *CEBPA* (only tested in patients with normal karyotype AML)	2 out of 20
*RUNX1* and *TP53* analysis according to ELN 2017 classification	15 (30%)
*RUNX1* mutated AML	2 out of 15
*TP53* mutated AML	1 out of 15
Hematologic malignancy	Not including blood count changes
Any Family member	8 (16%)
1st grade relative	2 (4%)
Solid malignancy	
Any family member	42 (84%)
1st grade relative	31 (62%)

**Fig 1:**
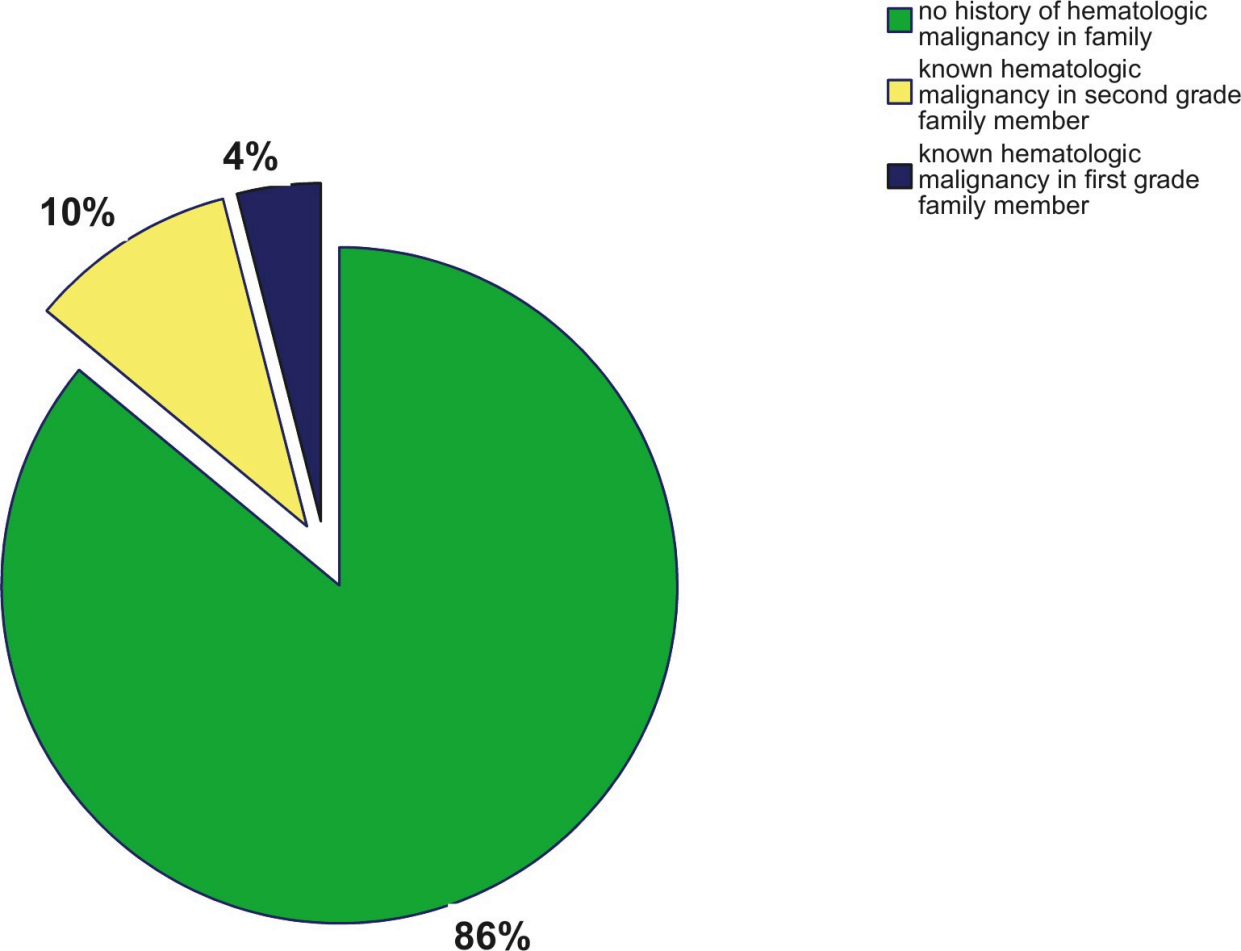
Frequency of hematologic malignancies in family history of 50 AML patients.

8/50 (16%) patients had family members with hematologic malignancies. [Table 2, Fig 1] One patient had two family members affected with hematologic malignancies. There was one case of Langerhans cell histiocytosis, one case of Burkitt lymphoma, one case of secondary AML, 4 cases of non-specified leukemia and 2 cases of non-specified lymphoma. 2/50 (4%) patients reported hematologic malignancies (secondary AML and non-specified leukemia) in one of their first-degree relatives (parents, children, siblings). Two patients had each a family member with known alterations in blood counts (platelets or white blood cells).

**Table 2:** Analysis of family history in 50 patients with acute myeloid leukemia.

Pat	Sex	Age at initial diagnosis in decade	Diagnosis	hematologic disease in family history, kinship degree, sex	solid malignancy in family history, kinship degree, sex	Accuracy compared with family history at admission at initial diagnosis	Inclusion Criteria for HNPCC or BRCA Testing or cancer risk assessment*
1	f	60–70	sAML	-	1) colon cancer, first degree, m	not obtained	
2	m	50–60	sAML	-	-	complete	
3	m	60–70	sAML	A)langerhans-cell histiocytosis, second	1) renal cancer, first, m	Initial diagnosis in external hospital	cancer risk assessment
					2) colon cancer, first, f		
					3) stomach cancer, second, m		
4	m	50–60	AML	A) sAML, first, f	1)urogenital cancer, first, m	incomplete: B), 1) missing	cancer risk assessment
				B)blood count changes, first,f			
5	m	60–70	AML	-	1)pancreatic cancer, first, m	incomplete: 3) missing	
					2) cervical cancer, first, f		
					3) cervical cancer, second, f		
6	m	50–60	AML	-	1) lung cancer, first, f	initial diagnosis in external hospital	
7	f	60–70	AML	-	1) breast cancer, first, f	NA	
8	m	40–50	AML	-	1) colon cancer, first, m	complete	
9	f	30–40	AML	-	1) breast cancer, second, f	complete	
					2) ENT cancer, second, m		
10	m	70–80	AML	-	1) colon cancer, second, f	incomplete: 1) missing	
11	m	70–80	sAML	-	1) colon cancer, first, f	no patient history (ICU)	HNPCC Amsterdam II criteria
					2) colon cancer, second, f		
					3) stomach cancer, second, m		
					4) stomach cancer, second, f		
12	f	70–80	AML	-	1) brain tumor, first, f	incomplete: 2) missing	
					2) colon cancer, third, m		
13	m	70–80	sAML	-	1) prostate cancer, first, m	no patient history (ICU)	
					2)unspecified cancer, third, m		
14	m	60–70	sAML	-	1) breast cancer, first, f	incomplete: 2), 3) missing	
					2)unspecified cancer, second,m		
					3)unspecified cancer, second, f		
15	m	20–30	AML	-	1) breast cancer, first, f	complete	
16	m	70–80	sAML	-	-	complete	
17	m	70–80	sAML	-	1) lung cancer, first, f	incomplete: 1) missing	
18	m	60–70	sAML	-	1) testicular cancer, first, m,	incomplete: 2) missing	
					2) colon cancer, second, f		
19	f	30–40	AML	A)leukemia/lymphoma, second,f	1) testicular cancer, first, m	incomplete: A), B) missing	
				B) Burkitt lymphoma, third, m	2) lung cancer, second, f		
				-	3) lung cancer, second, f		
20	m	60–70	AML	-	1) cervical cancer, first, f	not obtained	
					2) stomach cancer, first, m		
					3) renal cancer, second, f		
21	f	40–50	AML	-	1) lung cancer, second, m	incomplete: 1), 2) missing	
					2)unspecified cancer, second, f		
22	f	50–60	AML	A) lymphoma, second, f	1) lung cancer, first, m	no patient history (ICU)	cancer risk assessment
					2) lung cancer, first, m		
					3) cervical cancer, first, f		
					4) breast cancer, second, f		
23	m	60–70	AML	-	1)urogenital cancer, second, f	complete	
24	m	30–40	AML	-	1) colon cancer, second, f	incomplete: 1) missing	
25	m	40–50	AML	-	1) prostate cancer, first, m	not obtained	
26	f	30–40	AML	-	-	not obtained	
27	m	40–50	AML	A)blood count changes, first, m	1) renal cancer, first, m	incomplete: A) missing	cancer risk assessment
					2) brain tumor, first, m		
28	f	60–70	sAML	-	-	complete	
29	f	70–80	AML	-	1) ENT tumor, first, f	complete	
					2) breast cancer, first, f		
					3) colon cancer, second, f		
					4) breast cancer, second, f		
30	f	60–70	AML	-	1) liver cancer, first, m	incomplete: 1) missing	
31	m	40–50	AML	-	1) colon cancer, first, f	no patient history (ICU)	
					2)unspecified cancer, second, f		
32	m	60–70	AML	-	1)unspecified cancer, first, m	not obtained	
33	f	20–30	AML	-	1) breast cancer, second, f	incomplete: 1), 2) missing	*BRCA* testing indicated
					2) prostate cancer, second, m		
					3) breast cancer, second, f		
					4)urogenital cancer, second, f		
34	f	30–40	AML	-	1) bladder cancer, second, m	incomplete: 1) missing	
35	m	50–60	sAML	-	1)pancreatic cancer, first, f	incomplete: 1), 2), 3) missing	
					2) prostate cancer, first, m		
					3) lung cancer, second, m		
36	m	20–30	AML	-	1) bone cancer, second, m	initial diagnosis in external hospital	
37	m	50–60	AML	-	1) ENT tumor, first, m	discrepant	
38	m	40–50	AML	-	1) brain tumor, first, f	incomplete: 2), 3), 4) missing	
					2) breast cancer, second, f		
					3)unspecified cancer,second, m		
					4)unspecified cancer, second, f		
					5) prostate cancer, second, m		
39	m	80–90	AML	-	1)pancreatic cancer, first, f	incomplete: 1) missing	
					2) breast cancer, first, f		
40	m	70–80	AML	-	1) stomach cancer, second, m	discrepant	
					2) stomach cancer, second, m		
41	m	50–60	AML	-	-	not obtained	
42	f	60–70	sAML	-	-	initial diagnosis in external hospital	
43	m	60–70	AML	A) leukemia, first, f	-	initial diagnosis in external hospital	
44	f	50–60	sAML	A) leukemia, second, f	1)urogenital cancer, first, m	initial diagnosis in external hospital	
45	f	30–40	AML	-	1) colon cancer, second, f	no patient history	
46	m	20–30	AML	-	-	no patient history	
47	m	60–70	sAML	-	1) lung cancer, first, m	not obtained	
					2)unspecified cancer, first, f		
48	m	60–70	AML	A) leukemia, second, m	1) skin cancer, first, f	incomplete: A), 1), 3), 4) missing	cancer risk assessment
					2)urogenital cancer, first, f		
					3) breast cancer, first, f		
					4) breast cancer, second, f		
49	m	50–60	AML	A) leukemia, second, f	1)breast/urogenital cancer, first, f	incomplete: A), 1), 4), 5) missing	*BRCA* testing indicated cancer risk assessment
					2) breast cancer, first, f		
					3) breast cancer, first, f		
					4) colon cancer, second, m		
					5) colon cancer, second, m		
50	m	60–70	AML	-	1) lung cancer, second, m	Incomplete: 1) missing	

m male, f female, AML de-novo AML, sAML secondary AML, NA not available

*cancer risk assessment as suggested by Churpek et al.[15]

20/50 (40%) patients have a normal karyotype AML. Of these 20 patients 2 (14%) have AML with biallelic *CEBPA* mutation. This is a known mutation in familial AML cases. However, both patients with biallelic *CEPBA* AML in our cohort do not show a higher cancer risk in their family history.

As the recent ELN classification of 2017 [5] has added *RUNX1* and *TP53*-mutated AML to the adverse risk group, routine diagnostics for these genes were performed for patients first diagnosed after January 2017. In our study group 15 patients were diagnosed after this date. Of these 15 AML patients one patient has a *TP53*-mutated AML (7%) without any known malignancies in his family and two patients have a *RUNX1*-mutated AML (14%) of which one patient has a second grade family member with a lymphoma.

**Fig 2:**
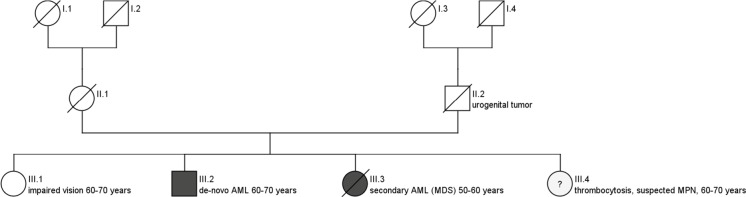
Schematic pedigree of AML patient with affected first degree family members.

In Fig 2, the simplified pedigree of a AML patient with one first grade family member who had a hematologic malignancy and another first grade family member with a suspected hematologic malignancy is shown.

**Fig 3:**
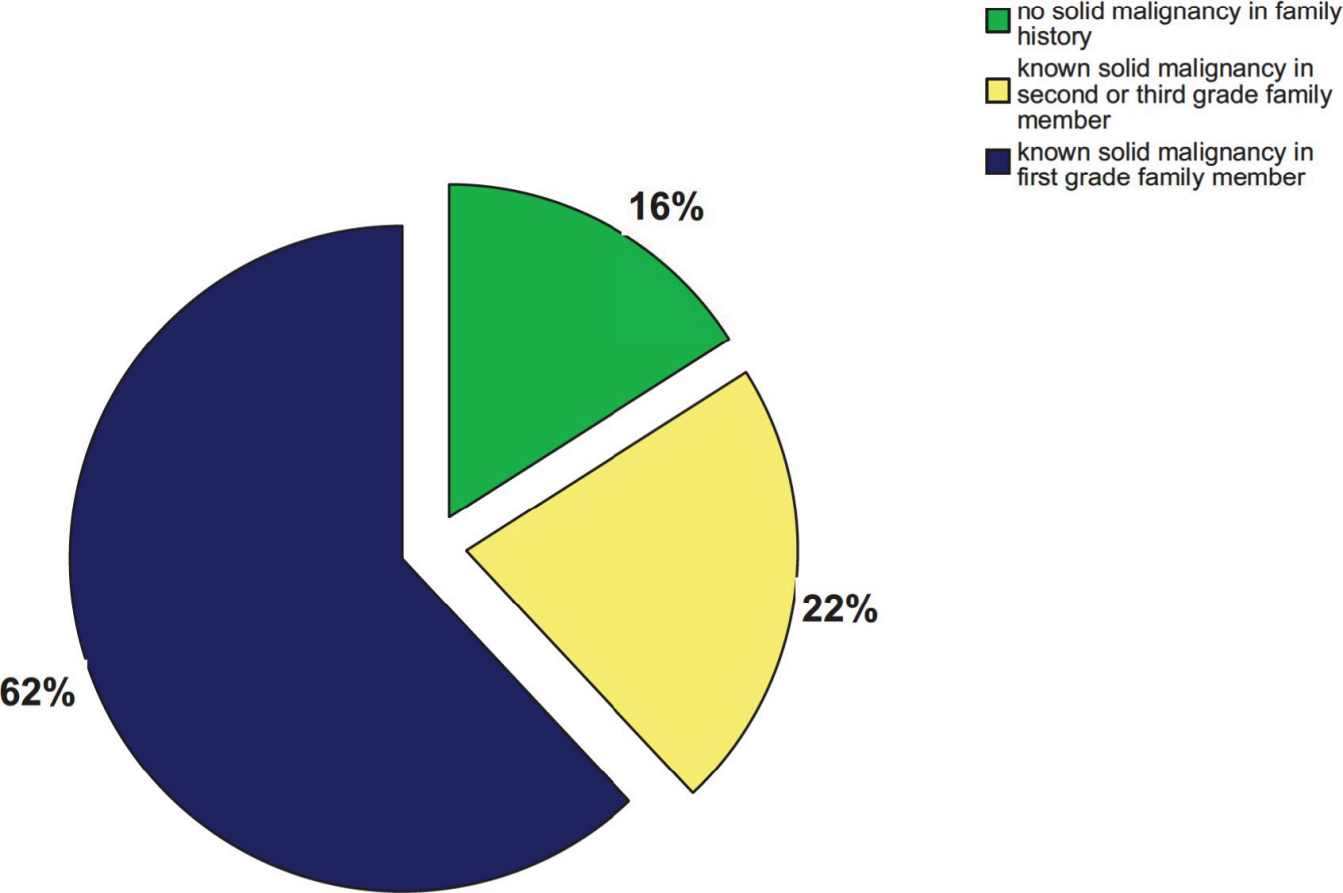
Frequency of solid malignancies in family history of 50 AML patients.

84% (42/50) of the patients had relatives with solid malignancies. [Table 2, Fig 3]. 5 AML patients had one first-degree relative with colon cancer, 7 patients had at least one first-grade relative with breast cancer, of which in one case a patient had 3 first-degree relatives with breast cancer. 4 patients had at least one first-grade relative with lung cancer.

One patient met the Bethesda criteria for HNPCC and two patients fulfilled the criteria for recommendation of genetic testing for hereditary breast cancer in their families.

**Fig 4:**
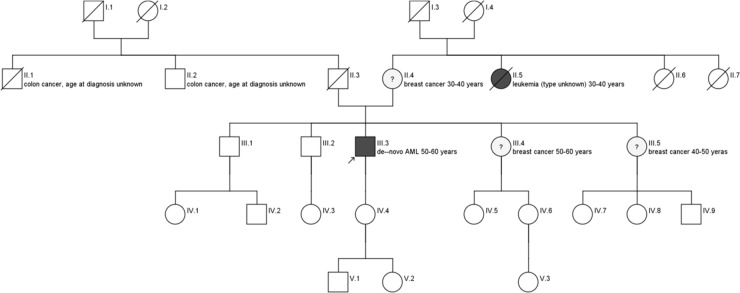
Schematic pedigree of AML patient with high risk constellation for familial breast cancer.

In Fig 4 the simplified pedigree of a male AML patient who meets the criteria for familial breast cancer is shown.

In a review article by Churpek et al. the authors suggest that patients with AML should be referred for comprehensive cancer risk assessment if the following criteria apply: A family history of MDS/AML or aplastic anaemia, early onset cancers of any type, multiple close relatives with cancer and/or a personal or family history of low blood counts, bleeding diathesis, skin or nail abnormalities, unexplained liver disease, pulmonary fibrosis or alveolar proteinosis, limb anomalies, lymphedema or immune deficiencies/atypical infections. [15]

When applying these criteria to our 50 AML patients and defining the criteria of “multiple close relatives with cancer” as more than 2 family members of first degree with cancer, then 6 of 50 (12%) patients fulfil these and should be referred for comprehensive cancer risk assessment.

None of the 50 patients in our cohort reported a family member with a known hemophilia, von Willebrand disease or other clinical relevant bleeding disorder.

We were able to analyze 29 pairs of family histories taken at first admission in our center in clinical routine compared with the pedigrees of this study. 28% (8/29) percent of the pairs were completely identical in regard to information about hematologic and solid malignancies in the family. In two cases the information was discrepant, of which in one case the tumor type was discrepant and in the other case the information about a solid cancer in a first-degree relative was not mentioned in the study pedigree, but two solid cancer cases in second-degree relatives, that were not mentioned in the clinical routine family history. Regarding the family histories of the 4 patients with hematologic malignancies in the family and a comparable family history at initial admission, only in one patient was the information of the hematologic malignancy obtained. In the other three patients, though a family history was obtained, the information about hematologic malignancies in the family was missed.

## Discussion

The aim of obtaining the family history of oncological patients is to identify patients with a genetic predisposition or patients at risk of second malignancies. The method of a pedigree interview is easy to apply, but typically taking 15–30 minutes it is not always feasible in the routine clinical practice. [19]

Missing information on families with a genetic tumor predisposition can prevent adequate counseling. Furthermore it can delay the search for a stem cell donor for allogeneic stem cell transplantation or even lead to the choice of a family donor with a possible germline predisposition.

In the clinical routine it is not always possible to take a detailed and thorough family history, for example due to lack of time, or because of cognitive deficits of the patient. The minimum family history should however encompass the cancer type and age at diagnosis of all first-degree (parents, children, siblings) and second-degree (grandparents, grandchildren, uncles, aunts, nieces, nephews, half-siblings) relatives with differentiation in maternal and paternal lineage as recommended for all cancer patients by the American Society of Clinical Oncology. [6] If possible, third-degree family members should also be included to provide a better overview of the family history.

Genetic predisposition to myeloid neoplasms [1, 20, 21, 22, 23] becomes more and more important to the clinician and more focus and emphasis must be put in taking the patient’s full history including a detailed family history at first admission, as this might have consequences for the patient and his family in screening family members for possible stem cell donations. Also, if the family history results in a suspected familial predisposition for hematologic disorders and other cancer syndromes, a human genetic counseling should be strongly recommended to the patient.

Our data suggest that the frequency of hematologic disorders in families of patients with myeloid neoplasms is higher than seen in the clinical routine. Of 50 AML patients 16% have at least one family member with a hematologic malignancy and 4% report an affected first grade relative. Furthermore we found solid malignancies in family members of first degree in 62% of the 50 AML patients.

Of note, in our cohort of 50 patients we identified two AML patients without history of breast cancer who met the criteria for hereditary breast cancer in their families. Especially in male family members of such families sometimes the indications for this cancer predisposition can be overseen, as they only seldom show the classic entities. Therefore especially in male AML patients with breast cancer in the family one should be careful to consider germline *BRCA* mutations as *BRCA1* and *BRCA2* mutations predispose to an increased risk for a broad spectrum of cancers, especially stomach, pancreas, prostate and colon cancer, but can also be associated with a higher occurrence of leukemia [8].

Moreover we identified one AML patient who met the Bethesda guidelines criteria for HNPCC in his family. There are several reported families with mismatch repair deficiency syndrome that show a predisposition to develop hematologic malignancies, often in childhood. [11, 24] These findings show that when obtaining the family history of hematologic patients there should also be a clinical awareness for these cancer syndromes, as it is relevant for genetic counseling and preventive measures as well as for a potential stem cell family donor search.

Another interesting finding in our study was that the accuracy of the family history taken at first admission in the chart review compared with the pedigree interview approach was only 28%. Our best explanation for this phenomenon is the thorough and time-intensive interview in this study with approximately 30 minutes per patients exclusively for the family history, which cannot be paralleled with the clinical routine.

In 7/37 (19%) of the medical charts a family cancer history was absent. A study of Carney et al. compared the association between a documented family history of cancer and the frequency of going to screening for breast and colorectal cancer. 44% percent of the charts of female patients and 56% of the charts of male patients contained no documentation of a family cancer history having been taken. Interestingly the absence of any recorded family cancer history was associated with a decreased likelihood for cancer screening. [25] Van Dijk et al. investigated the frequency of an adequate screening for HNPCC criteria by taking the family history in patients with colorectal carcinoma. They screened the diagnostic work-up of 224 patients and found out that a complete family history was recorded for only 16%. [26]

A noteworthy study with a large cohort of 1112 patients with epithelial ovarian cancer assessed the family history for breast and ovarian cancer found in the medical records. In 41% of the patients, an adequate family history for breast and ovarian cancer was taken. Remarkably, in this study younger age, an academic hospital and having undergone surgery and/or chemotherapy were factors associated with taking an adequate family history. In our study the median age was 59.5, which might contribute to the smaller percentage of adequate family history found in the medical records at initial admission, also the aforementioned study only evaluated the family history for specific cancer types, not all malignancies. [27]

As concluded in an interesting study by Frezzo et al. that assessed the genetic family history as a risk assessment tool in internal medicine, the use of the genetic three-generation pedigree instead of the conventional medical history should be considered. [28]

In a Scottish study even in face to face interviews the accuracy of recalling and reporting a complete and accurate family history of colorectal cancer in patients with this disease, a positive family history was significantly underreported compared to the data of the cancer registry. This can lead to the assumption that there is a substantial high number of unreported cases of positive family cancer history in the clinical routine. [29]

Son et al. were able to show that the accuracy of the pedigree and family cancer history increases when patients are interviewed several times. [30] This emphasizes the point that in cancer patients not only at the time of first diagnosis the family history is crucial but also throughout the following time. [15]

We acknowledge some restrictions of this study. The data acquired in this study were based on the patients’ recall of their family history leading to a recall bias.

Moreover a considerable number of patients were not able to remember neither the exact type of the cancer nor the age at disease onset in their relatives. Also we did not compare the family history in the chart of patients who were transferred to the stem cell transplantation checkup examination, but only at initial admission or return visit to the normal ward.

Also there are so called cancer families with a high prevalence of malignant diseases among family members, in which no inherited genetic mutations can be found. In these cases, extrinsic cancer risks, such as a shared environment or tobacco use are considered to be the cause. [31]

This study was based on a relatively small number of patients. Investigating larger populations with molecular genetic information may lead to further information about the frequency of cancer predisposition in AML patients. Genetic counseling was recommended to every patient with indication for genetic risk as recommended by the ASCO guidelines [6], the uptodate guidelines on familial acute leukemia [32] and myelodysplastic syndromes as well as the German guideline for genetic counseling by the German Society for Human Genetics. [33]

We are optimistic that in the future methods to acquire a thorough three generation family history in an easy, quick and accessible manner, for example in digital format, can be implemented in the broad clinical routine in oncologic care center. Several tools have already been developed and used. [34] Also there are publicly available tools like My Family Health Portrait for patients to draw their pedigree and learn about possible risks for common conditions. [35]

To the best of our knowledge, this is the first descriptive study to evaluate the frequency of hematologic and solid malignancies in the family histories of AML patients. Taken together, in the last years great progress has been made in the research of familial leukemia. This notion has to successfully transfer to the clinical routine with a greater emphasis and focus on the family history and comprehension of a pedigree interview in leukemia patients in order to recommend genetic counseling to patients at risk and to facilitate the search for possible stem cell donors.

## Supporting information

S1 Questionnaire Family history of patients with AML (acute myeloid leukemia). (DOCX)
