# Supplementary material for: Frequency of hematologic and solid malignancies in the family history of 50 patients with acute myeloid leukemia – a single center analysis
Source: PLoS One. 2019 Apr 18;14(4):e0215453. doi: 10.1371/journal.pone.0215453 (PMC6472770; doi:10.1371/journal.pone.0215453)
Supplement: S1 Questionnaire — (DOCX) [file pone.0215453.s001.docx]

S1. Questionnaire: Family history of patients with AML (acute myeloid leukemia)

| Name |  | Surname |  |
| --- | --- | --- | --- |
| Date of birth |  | Date: |  |
| Date of first  diagnosis: |  | Current  therapy: |  |

| 1) Father (date of birth_______) age at first diagnosis: | | | |  |  |
| --- | --- | --- | --- | --- | --- |
|  | □ | colorectal cancer | □ leukemia (exact type known?) |  |  |
|  | □ | lung cancer | □ lymphoma (exact type known?) |  |  |
|  | □ | pancreatic cancer | □ other hematologic malignancy? Please specify: |  |  |
|  | □ | prostate cancer | □ blood count changes: |  |  |
|  | □ | no malignancy known | □ bleeding disorder: |  |  |
|  |  |  | □ skin disorder, please specify: |  |  |
|  | □ | other malignancies, please specify: | |  |  |
|  |  |  | |  |  |

| 2) Mother (date of birth:________) age at first diagnosis: | | | |  |  |
| --- | --- | --- | --- | --- | --- |
|  | □ | colorectal cancer | □ leukemia (exact type known?) |  |  |
|  | □ | lung cancer | □ lymphoma (exact type known?) |  |  |
|  | □ | pancreatic cancer | □ other hematologic malignancy? Please specify: |  |  |
|  | □ | breast cancer | □ blood count changes: |  |  |
|  | □ | no malignancy known | □ bleeding disorder: |  |  |
|  |  |  | □ skin disorder, please specify: |  |  |
|  | □ | other malignancies, please specify: | |  |  |
|  |  |  | |  |  |

| 3) Child (date of birth:________) □ son □daughter age at first diagnosis: | | | |
| --- | --- | --- | --- |
|  | □ | colorectal cancer | □ leukemia (exact type known?) |
|  | □ | lung cancer | □ lymphoma (exact type known?) |
|  | □ | pancreatic cancer | □ other hematologic malignancy? Please specify: |
|  | □ | breast cancer | □ blood count changes: |
|  | □ | no malignancy known | □ bleeding disorder: |
|  |  |  | □ skin disorder, please specify: |
|  | □ | other malignancies, please specify: | |
|  |  |  | |

| 4) Child (date of birth:________) □ son □daughter age at first diagnosis: | | | |
| --- | --- | --- | --- |
|  | □ | colorectal cancer | □ leukemia (exact type known?) |
|  | □ | lung cancer | □ lymphoma (exact type known?) |
|  | □ | pancreatic cancer | □ other hematologic malignancy? Please specify: |
|  | □ | breast cancer | □ blood count changes: |
|  | □ | prostate cancer | □ bleeding disorder: |
|  | □ | no malignancy known | □ skin disorder, please specify: |
|  | □ | other malignancies, please specify: | |
|  |  |  | |

| 5) Child (date of birth:________) □ son □daughter age at first diagnosis: | | | |
| --- | --- | --- | --- |
|  | □ | colorectal cancer | □ leukemia (exact type known?) |
|  | □ | lung cancer | □ lymphoma (exact type known?) |
|  | □ | pancreatic cancer | □ other hematologic malignancy? Please specify: |
|  | □ | breast cancer | □ blood count changes: |
|  | □ | prostate cancer | □ bleeding disorder: |
|  | □ | no malignancy known | □ skin disorder, please specify: |
|  | □ | other malignancies, please specify: | |
|  |  |  | |

| 5) paternal grandfather (date of birth:________) age at first diagnosis: | | | |
| --- | --- | --- | --- |
|  | □ | colorectal cancer | □ leukemia (exact type known?) |
|  | □ | lung cancer | □ lymphoma (exact type known?) |
|  | □ | pancreatic cancer | □ other hematologic malignancy? Please specify: |
|  | □ | prostate cancer | □ blood count changes: |
|  | □ | no malignancy known | □ bleeding disorder: |
|  |  |  | □ skin disorder, please specify: |
|  | □ | other malignancies, please specify: | |
|  |  |  | |

| 6) paternal grandmother (date of birth:________) age at first diagnosis: | | | |
| --- | --- | --- | --- |
|  | □ | colorectal cancer | □ leukemia (exact type known?) |
|  | □ | lung cancer | □ lymphoma (exact type known?) |
|  | □ | pancreatic cancer | □ other hematologic malignancy? Please specify: |
|  | □ | breast cancer | □ blood count changes: |
|  | □ | no malignancy known | □ bleeding disorder: |
|  |  |  | □ skin disorder, please specify: |
|  | □ | other malignancies, please specify: | |
|  |  |  | |

| 7) maternal grandfather (date of birth:________) age at first diagnosis: | | | |
| --- | --- | --- | --- |
| □ | colorectal cancer | □ leukemia (exact type known?) |  |
| □ | lung cancer | □ lymphoma (exact type known?) |  |
| □ | pancreatic cancer | □ other hematologic malignancy? Please specify: |  |
| □ | prostate cancer | □ blood count changes: |  |
| □ | no malignancy known | □ bleeding disorder: |  |
|  |  | □ skin disorder, please specify: |  |
| □ | other malignancies, please specify: | |  |
|  |  | |  |

| 8) maternal grandmother (date of birth:________) age at first diagnosis: | | | |
| --- | --- | --- | --- |
|  | □ | colorectal cancer | □ leukemia (exact type known?) |
|  | □ | lung cancer | □ lymphoma (exact type known?) |
|  | □ | pancreatic cancer | □ other hematologic malignancy? Please specify: |
|  | □ | breast cancer | □ blood count changes: |
|  | □ | no malignancy known | □ bleeding disorder: |
|  |  |  | □ skin disorder, please specify: |
|  | □ | other malignancies, please specify: | |
|  |  |  | |

| 9) sibling (date of birth:________) □ brother □ sister age at first diagnosis: | | | |
| --- | --- | --- | --- |
|  | □ | colorectal cancer | □ leukemia (exact type known?) |
|  | □ | lung cancer | □ lymphoma (exact type known?) |
|  | □ | pancreatic cancer | □ other hematologic malignancy? Please specify: |
|  | □ | breast cancer | □ blood count changes: |
|  | □ | prostate cancer | □ bleeding disorder: |
|  | □ | no malignancy known | □ skin disorder, please specify: |
|  | □ | other malignancies, please specify: | |
|  |  |  | |

| 10) sibling (date of birth:________) □ brother □ sister age at first diagnosis: | | | |
| --- | --- | --- | --- |
|  | □ | colorectal cancer | □ leukemia (exact type known?) |
|  | □ | lung cancer | □ lymphoma (exact type known?) |
|  | □ | pancreatic cancer | □ other hematologic malignancy? Please specify: |
|  | □ | breast cancer | □ blood count changes: |
|  | □ | prostate cancer | □ bleeding disorder: |
|  | □ | no malignancy known | □ skin disorder, please specify: |
|  | □ | other malignancies, please specify: | |
|  |  |  | |

| 11) sibling (date of birth:________) □ brother □ sister age at first diagnosis: | | | |
| --- | --- | --- | --- |
|  | □ | colorectal cancer | □ leukemia (exact type known?) |
|  | □ | lung cancer | □ lymphoma (exact type known?) |
|  | □ | pancreatic cancer | □ other hematologic malignancy? Please specify: |
|  | □ | breast cancer | □ blood count changes: |
|  | □ | prostate cancer | □ bleeding disorder: |
|  | □ | no malignancy known | □ skin disorder, please specify: |
|  | □ | other malignancies, please specify: | |
|  |  |  | |

| 11) siblings of father(date of birth:________) □ uncle □ aunt age at first diagnosis: | | | |
| --- | --- | --- | --- |
|  | □ | colorectal cancer | □ leukemia (exact type known?) |
|  | □ | lung cancer | □ lymphoma (exact type known?) |
|  | □ | pancreatic cancer | □ other hematologic malignancy? Please specify: |
|  | □ | breast cancer | □ blood count changes: |
|  | □ | prostate cancer | □ bleeding disorder: |
|  | □ | no malignancy known | □ skin disorder, please specify: |
|  | □ | other malignancies, please specify: | |
|  |  |  | |

| 12) siblings of father(date of birth:________) □ uncle □ aunt age at first diagnosis: | | | |
| --- | --- | --- | --- |
|  | □ | colorectal cancer | □ leukemia (exact type known?) |
|  | □ | lung cancer | □ lymphoma (exact type known?) |
|  | □ | pancreatic cancer | □ other hematologic malignancy? Please specify: |
|  | □ | breast cancer | □ blood count changes: |
|  | □ | prostate cancer | □ bleeding disorder: |
|  | □ | no malignancy known | □ skin disorder, please specify: |
|  | □ | other malignancies, please specify: | |
|  |  |  | |

| 13) siblings of father(date of birth:________) □ uncle □ aunt age at first diagnosis: | | | |
| --- | --- | --- | --- |
|  | □ | colorectal cancer | □ leukemia (exact type known?) |
|  | □ | lung cancer | □ lymphoma (exact type known?) |
|  | □ | pancreatic cancer | □ other hematologic malignancy? Please specify: |
|  | □ | breast cancer | □ blood count changes: |
|  | □ | prostate cancer | □ bleeding disorder: |
|  | □ | no malignancy known | □ skin disorder, please specify: |
|  | □ | other malignancies, please specify: | |
|  |  |  | |

| 14) siblings of mother(date of birth:________) □ uncle □ aunt age at first diagnosis: | | | |
| --- | --- | --- | --- |
|  | □ | colorectal cancer | □ leukemia (exact type known?) |
|  | □ | lung cancer | □ lymphoma (exact type known?) |
|  | □ | pancreatic cancer | □ other hematologic malignancy? Please specify: |
|  | □ | breast cancer | □ blood count changes: |
|  | □ | prostate cancer | □ bleeding disorder: |
|  | □ | no malignancy known | □ skin disorder, please specify: |
|  | □ | other malignancies, please specify: | |
|  |  |  | |

| 15) siblings of mother(date of birth:________) □ uncle □ aunt age at first diagnosis: | | | |
| --- | --- | --- | --- |
|  | □ | colorectal cancer | □ leukemia (exact type known?) |
|  | □ | lung cancer | □ lymphoma (exact type known?) |
|  | □ | pancreatic cancer | □ other hematologic malignancy? Please specify: |
|  | □ | breast cancer | □ blood count changes: |
|  | □ | prostate cancer | □ bleeding disorder: |
|  | □ | no malignancy known | □ skin disorder, please specify: |
|  | □ | other malignancies, please specify: | |
|  |  |  | |

| 16) siblings of mother(date of birth:________) □ uncle □ aunt age at first diagnosis: | | | |
| --- | --- | --- | --- |
|  | □ | colorectal cancer | □ leukemia (exact type known?) |
|  | □ | lung cancer | □ lymphoma (exact type known?) |
|  | □ | pancreatic cancer | □ other hematologic malignancy? Please specify: |
|  | □ | breast cancer | □ blood count changes: |
|  | □ | prostate cancer | □ bleeding disorder: |
|  | □ | no malignancy known | □ skin disorder, please specify: |
|  | □ | other malignancies, please specify: | |
|  |  |  | |

| 17) other relative (date of birth:________) age at first diagnosis: | | | |
| --- | --- | --- | --- |
| kinship degree?______________________ □ maternal □ paternal | | | |
|  | □ | colorectal cancer | □ leukemia (exact type known?) |
|  | □ | lung cancer | □ lymphoma (exact type known?) |
|  | □ | pancreatic cancer | □ other hematologic malignancy? Please specify: |
|  | □ | breast cancer | □ blood count changes: |
|  | □ | prostate cancer | □ bleeding disorder: |
|  | □ | no malignancy known | □ skin disorder, please specify: |
|  | □ | other malignancies, please specify: | |
|  |  |  | |

| 18) other relative (date of birth:________) age at first diagnosis: | | | |
| --- | --- | --- | --- |
| kinship degree?______________________ □ maternal □ paternal | | | |
|  | □ | colorectal cancer | □ leukemia (exact type known?) |
|  | □ | lung cancer | □ lymphoma (exact type known?) |
|  | □ | pancreatic cancer | □ other hematologic malignancy? Please specify: |
|  | □ | breast cancer | □ blood count changes: |
|  | □ | prostate cancer | □ bleeding disorder: |
|  | □ | no malignancy known | □ skin disorder, please specify: |
|  | □ | other malignancies, please specify: | |
|  |  |  | |

| 19) other relative (date of birth:________) age at first diagnosis: | | | |
| --- | --- | --- | --- |
| kinship degree?______________________ □ maternal □ paternal | | | |
|  | □ | colorectal cancer | □ leukemia (exact type known?) |
|  | □ | lung cancer | □ lymphoma (exact type known?) |
|  | □ | pancreatic cancer | □ other hematologic malignancy? Please specify: |
|  | □ | breast cancer | □ blood count changes: |
|  | □ | prostate cancer | □ bleeding disorder: |
|  | □ | no malignancy known | □ skin disorder, please specify: |
|  | □ | other malignancies, please specify: | |
|  |  |  | |

| 20) other relative (date of birth:________) age at first diagnosis: | | | |
| --- | --- | --- | --- |
| kinship degree?______________________ □ maternal □ paternal | | | |
|  | □ | colorectal cancer | □ leukemia (exact type known?) |
|  | □ | lung cancer | □ lymphoma (exact type known?) |
|  | □ | pancreatic cancer | □ other hematologic malignancy? Please specify: |
|  | □ | breast cancer | □ blood count changes: |
|  | □ | prostate cancer | □ bleeding disorder: |
|  | □ | no malignancy known | □ skin disorder, please specify: |
|  | □ | other malignancies, please specify: | |
|  |  |  | |
